# Editorial Brevities

**Published:** 1904-02

**Authors:** 


### EDITORIAL BREVITIES.

DR. C. F. BENSON has been elected president of the Atlanta Board of Health.

---

DR. W. B. ARMSTRONG has been elected president of the Board of Trustees of Grant Park.

---

DR. CYRUS W. STRICKLER has been elected a member of the Atlanta Board of Health in place of Dr. E. H. Richardson, whose term of office expired.

---

THE death of Dr. W. C. Fisher, the county physician, of Bolton, which occurred in December, brought about some changes in this office. There are now two county physicians at a salary of $800 per annum. They are not required to devote their time exclusively to the county office. The newly elected physicians are Drs. E. D. Richardson and J. W. Hurt.

